# Critical appraisal of multidimensional CT measurements following acute open repair of type A aortic dissection

**DOI:** 10.1111/jocs.14446

**Published:** 2020-02-06

**Authors:** Ignas B. Houben, Theodorus M. J. van Bakel, Nicholas S. Burris, Frans L. Moll, Joost A. van Herwaarden, Himanshu J. Patel

**Affiliations:** ^1^ Department of Cardiac Surgery Frankel Cardiovascular Center, University of Michigan Health Center Ann Arbor Michigan; ^2^ Department of Vascular Surgery Frankel Cardiovascular Center, University of Michigan Health Center Ann Arbor Michigan; ^3^ Department of Radiology University of Michigan Health Center Ann Arbor Michigan; ^4^ Department of Vascular Surgery University Medical Center Utrecht Heidelberglaan The Netherlands

**Keywords:** 3D imaging, angiographic computed tomography, diameter, false lumen, interrater agreement, morphology, type A dissection

## Abstract

**Introduction:**

To identify patients with aneurysmal degeneration of the native aorta following type A aortic dissection (TAAD), reproducible serial measurements of aortic dimensions are critical. We used a systematic workflow for measuring aortic geometry following TAAD, using computed tomography angiography data, and test its reproducibility.

**Methods:**

The workflow for aortic measurements included centerline generation, luminal diameter, and area measurement at six anatomically defined locations along the aorta and luminal volumetric measurements in the descending aorta. Two independent observers measured the aortic geometry in 20 surgically repaired TAAD patients, preoperatively and at 3 months follow‐up. To test reproducibility, intraobserver and interobserver agreement scores were analyzed using a concordance correlation coefficient (CCC).

**Results:**

The interobserver agreement scores of the diameter, area, and volumetric measurements in the descending aorta were acceptable. The agreement scores of the area measurements were highest, with CCCs ranging from 0.909 to 0.984. Luminal diameter measurements scored lower than luminal area measurements and were least reproducible at the mid aortic arch (CCC < 0.886). Overall, intraobserver agreement scores were better than interobserver agreement scores (SD of mean difference was 1.89 vs 1.94 for intraobserver vs interobserver diameter measurements, and 0.61 vs 0.66 for area measurements).

**Conclusion:**

Although overall reproducibility was acceptable in descending aortic measurements, our results show that it remains challenging to reliably measure luminal diameters, compared with areas. To aid identification of early adverse remodeling following acute TAAD, novel two‐ and three‐dimensional measurement techniques are needed that capture locoregional changes in the false lumen and true lumen morphology more accurately.

## 1 INTRODUCTION

Currently, 80% to 90% of type A aortic dissection (TAAD) patients who make it to the hospital, survive the first 30 days following repair.^1^, ^2^ More than 10% of these patients will require surgical reintervention during follow‐up, most commonly due to adverse remodeling and dilation of the false lumen (FL).^3^, ^4^, ^5^ Recent registry data have shown that thoracic endovascular aortic repair (TEVAR) in the subacute phase following aortic dissection (2 weeks to 3 months) yields a lower mortality rate and significantly larger degree of positive aortic remodeling compared with endovascular repair in the chronic phase.^6^ This difference has been attributed to thickening and stiffening of the intimal flap over time,^7^ increasing the risk of endograft related complications.^8^ Following these results, early detection of adverse aortic remodeling is desirable to identify patients who will require aortic repair during follow‐up.^9^ Currently, diameter measurements of the aortic lumen are the mainstay measurement technique for the assessment of aortic geometry over time. Additionally, luminal area and volumetric measurements can be obtained from computed tomography angiography (CTA) image data. In recent years, various measurement techniques have been used to define endpoints in studies analyzing the effectiveness of different techniques for aortic repair.^10^, ^11^, ^12^, ^13^ However, these studies all use a different approach for measuring aortic morphology, and the interobserver and intraobserver agreement scores are not reported, making their reproducibility questionable.

To compare the results of different studies analyzing the morphologic changes following acute TAAD, a systematic workflow for measuring aortic morphology following TAAD is needed. In the present study, we used a systematic workflow for measuring aortic geometry following acute TAAD using two‐ and three‐dimensional measurement techniques, and test its reproducibility.

## 2 METHODS

Approval for this study was obtained from the institutional review board (University of Michigan, protocol number: HUM00061722; date of approval, 21 May 2012), the need for patient consent was waived. The University of Michigan's cardiac surgery database was retrospectively queried to identify 20 TAAD patients (DeBakey type I), who had available preoperative and 3‐month postoperative CTA examinations. CTA examinations were acquired on a multislice scanner after intravenous injection of 120 mL iopamidol intravenous contrast (Isoview 370; Bracco Diagnostics, Milan, Italy) and prospective reconstruction was performed in the mid‐diastolic phase (75% of the R‐R interval). The CTA image data were analyzed using automatic image processing tools in the software package Vitrea Core (Product Version 6.9.1; Vital Images Inc, Minnetonka, MN). All measurements were performed by two observers, TMJvB and IBH. For intraobserver measurements, an interval between measurements of at least 2 weeks was followed and scans were randomly reordered in between measurement intervals to avoid pattern recognition.

### 2.1 Image processing

**Figure 1:**
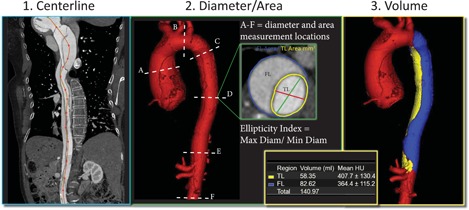
The workflow of our measurements depicting (1) manual centerline generation, (2) diameter and area measurements at six locations (A‐F), and (3) volumetric measurements using a vessel growth tool. FL, false lumen; TL, true lumen

In the following, the systematic workflow for measuring diameters, areas, and volumes in the regions of interest (ROIs) is reported. Figure 1 presents a visualization of the different steps in our workflow. First, CTA imaging data were imported in Vitrea Core. Then, a center line (CL) of the whole aorta, including FL and true lumen (TL), was manually generated starting at the level of the aortic valve and ending at the aortoiliac bifurcation. Subsequently, a curved multiplanar reformatted image was generated to confirm the central position of the CL within the total aortic perimeter. If needed, manual adjustments were made in areas of high tortuosity. Using planes perpendicular to the CL, ROIs were manually drawn to obtain diameter in mm and luminal area in cm^2^. ROIs were drawn delineating the total aortic lumen (outer wall of the aorta including both TL and FL), and the separate luminal areas. Intraluminal calcifications were included in the ROI. Ellipticity was defined as the largest diameter of the lumen divided by the smallest diameter. The abovementioned measurements were obtained in six locations along the CL: (a) the mid ascending aorta (midpoint between the aortic valve and the origin of the innominate artery); (b) the mid aortic arch (midpoint between left common carotid and subclavian arteries); (c) the proximal descending aorta (2 cm distal to distal end of the left subclavian artery [LSA]); (d) the mid descending aorta (10 cm distal to distal end of the LSA); (e) the distal descending aorta (2 cm proximal to proximal end of celiac trunk); and (f) infrarenal (1.5 cm distal to the most inferior renal artery). TL and FL volume measurements were obtained using a semiautomated threshold‐based segmentation tool with manual adjustments where needed. Areas of FL thrombus were included in the volume measurements. Both volumes were measured in the descending thoracic aorta, starting just distal to the origin of the LSA and ending just proximal to the origin of the celiac trunk.

### 2.2 Sample size calculation

Sample size calculations were based on a *ρ*0 (H0 lowest acceptable concordance correlation coefficient [CCC]) of 0.9 and a *ρ*1 (H1 expected outcome of CCC) of 0.95, yielding a required sample size of *n* = 32 observations using a significance (*α*) = .05 and a power (1 − *β*) of 0.80.^14^ In our case, 40 observed scans from 20 patients were used. As the morphology presurgical and postsurgical intervention affected the morphology significantly, we considered all 40 scans to be independent.

### 2.3 Statistical analysis

Normal distribution of the continuous data was tested using the Shapiro‐Wilk test. Logarithmic transformation was used to achieve normal distribution of the data where necessary. The 95% limits of agreement were defined as the mean difference ± repeatability coefficient (SD*1.96). These results were visually depicted using Bland‐Altman plots. A priori acceptable differences for diameter, luminal area, and volumetric measurements were added to the Bland‐Altman plots. These were respectively defined as 3 mm, 1 cm^2^, and 30 mL, based on clinical expertise and earlier work for type B aortic dissection (TBAD).^15^ Intraobserver and interobserver agreement were analyzed using a CCC.^16^ CCC values less than 0.90 were considered “poor agreement,” between 0.90‐0.95 “moderate,” 0.95‐0.99 “substantial,” and >0.99 “almost perfect” as described by Lin et al.^17^ Continuous data are presented using mean ± SD. *P* values < .05 were considered statistically significant. All tests were performed using SPSS version 24.0 (IBM SPSS Statistics, Armonk, NY).

## 3 RESULTS

**Figure 2:**
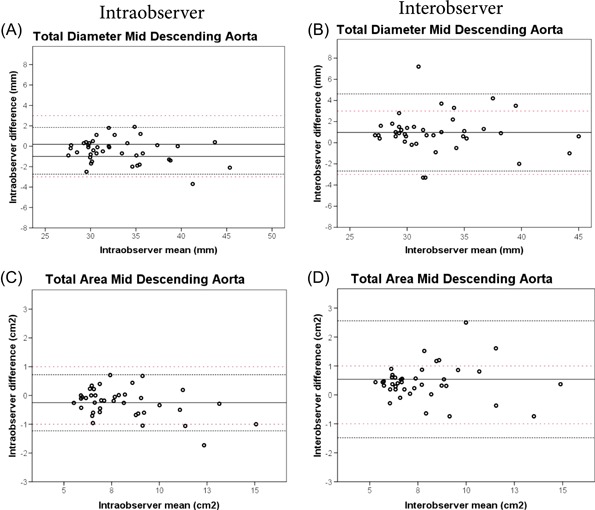
Intraobserver vs interobserver Bland‐Altman plots for maximum total aortic diameter measurements (A vs B) and for maximum total area measurements (C vs D) at the mid descending aorta. The solid black line represents the mean of all measurements paired. The black close dotted lines represent the limits of agreement and the red double spaced dotted line represents the a priori defined acceptable differences. The limits of agreement are defined within range of the a priori acceptable differences when the black dotted lines both do not exceed the red dotted lines. The variability is higher for interobserver (B, D) than for intraobserver (A, C) measurements

Among the 20 included patients, 10 were male (50%). The mean age was 60.4 ± 12.1 years. All acute TAAD patients received surgical repair within 14 days after onset of dissection. Surgery extended to zone 0 in 10 (50%) patients and to zone 1 in 10 (50%) patients and the ascending aortic graft ranged 24 to 30 mm in diameter. Mean follow‐up duration at the postoperative analysis was 104.0 ± 24.5 days. The combined diameter and area assessment over the dissected areas showed higher agreement for intraobserver (CCC = 0.894) vs interobserver (CCC = 0.881) measurements. Overall, intraobserver variability was lower than interobserver variability for diameter measurements (SD of mean difference of 1.89 vs 1.94 for intraobserver vs interobserver) and for area measurements (SD of mean difference of 0.61 vs 0.66). The difference between intraobserver and interobserver measurement variability is shown in Figure 2. The results of the interobserver reproducibility analysis will be the focus of the remainder of this study, as the higher degree of interobserver variability will drive the overall reliability of our measurement workflow in a typical clinical situation. The results of all intraobserver and interobserver measurement analyses are reported in the Supporting Information Data.

### 3.1 Aortic growth

Table 1 shows high variability in the luminal diameter growth assessment of the descending aorta, particularly on FL growth assessment in the proximal descending aorta, being −1.02 ± 14.41 mm. The FL area growth assessment in the proximal descending aorta is positive, showing 1.66 ± 1.23 cm^2^.

**Table 1:** Mean postoperative change on measurements of both observers combined

	Total diameter, mm	TL diameter, mm	FL diameter, mm	Total area, cm^2^	TL area, cm^2^	FL area, cm^2^	Total volume, mL	TL volume, mL	FL volume, mL
Proximal descending aorta	4.93 ± 5.17	2.31 ± 2.86	−1.02 ± 14.41	2.49 ± 2.91	0.94 ± 0.69	1.66 ± 1.23			
Mid descending aorta	3.11 ± 3.02	1.89 ± 1.58	2.70 ± 3.92	1.59 ± 1.56	0.07 ± 1.46	1.45 ± 2.29	40.33 ± 32.73	9.67 ± 22.67	30.66 ± 40.86
Distal descending aorta	2.19 ± 1.89	1.01 ± 2.00	1.93 ± 1.76	0.97 ± 0.81	0.18 ± 0.75	1.01 ± 1.36			
Infrarenal aorta^a^	1.43 ± 1.29	1.20 ± 1.52	1.26 ± 1.50	0.44 ± 0.61	0.29 ± 0.56	1.01 ± 1.19	N/A	N/A	N/A
Total descending aorta	3.41 ± 3.75	1.74 ± 2.25	1.16 ± 8.81	1.68 ± 2.03	0.46 ± 1.45	1.31 ± 2.64	N/A	N/A	N/A
Unrepaired segment growth	2.05 ± 4.10	1.13 ± 2.56	−3.63 ± 13.78	0.95 ± 2.12	0.78 ± 1.59	0.39 ± 2.89	N/A	N/A	N/A

*Note*: Continuous data are presented as the mean ± standard deviation.

Abbreviations: FL, false lumen; TL, true lumen.

^a^ Eight out of 20 growth moments excluded because of missing computed tomography data.

### 3.2 Aortic diameter and area agreement

**Figure 3:**
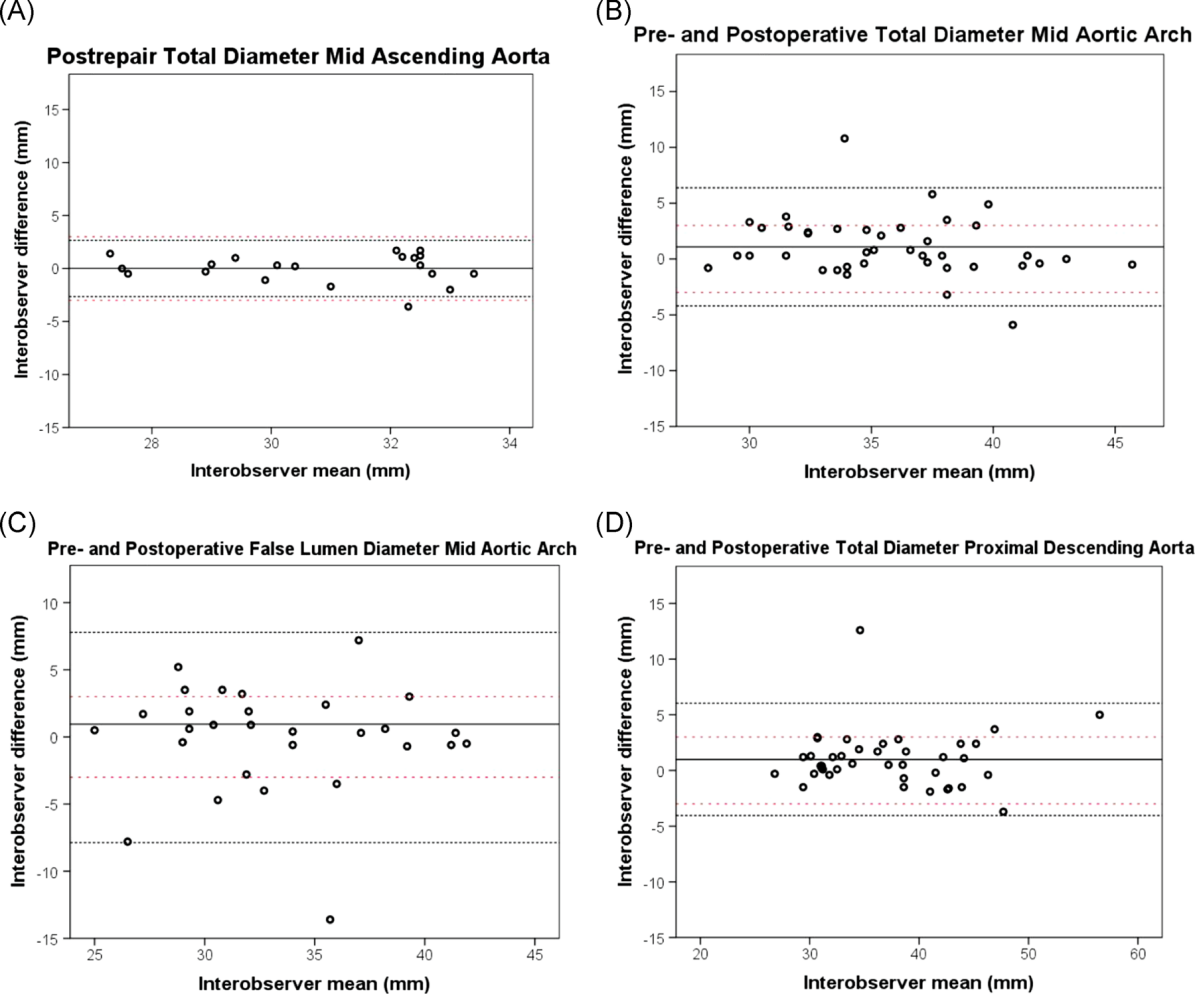
Interobserver Bland‐Altman plots for maximum diameter measurements at the (A) postrepair midascending aorta as a low‐error reference for comparison, (B) preoperative and postoperative mid aortic arch, (C) preoperative and postoperative false luminal mid aortic arch and (D) preoperative and postoperative proximal descending aorta. The solid black line represents the mean of all measurements paired. The black close dotted lines represent the limits of agreement and the red double spaced dotted line represents the a priori defined acceptable differences. The limits of agreement are defined within the range of the a priori acceptable differences when the black dotted lines both do not exceed the red dotted lines

A postrepair analysis of the ascending aorta revealed acceptable limits of agreement based on our a priori definitions (Figure 3A and Table 2). Since the repaired segment of the ascending aorta was expected to have stable diameters with good contrast enhancement and no FL, the Bland‐Altman plot was used as a baseline comparison (Figure 3A).

**Table 2:** Interobserver agreement for preoperative mid‐ascending aorta measurements

	Mean ± SD	95% limits of agreement	CCC	CCC agreement
Total diameter, mm	1.54 ± 2.04	−2.45, 5.53	0.903 (0.785, 0.958)	Moderate
TL max diameter, mm	0.31 ± 1.92	−3.45, 4.06	0.957 (0.897, 0.983)	Substantial
FL max diameter, mm	1.59 ± 2.06	−2.43, 5.62	0.893 (0.762, 0.954)	Fair
Total area, cm^2^	1.03 ± 0.89	−0.71, 2.77	0.953 (0.899, 0.979)	Substantial
TL area, cm^2^	0.15 ± 0.79	−1.41, 1.70	0.960 (0.903, 0.984)	Substantial
FL area, cm^2^	0.88 ± 0.92	−0.92, 2.68	0.955 (0.900, 0.980)	Substantial

*Note*: Continuous data are presented as the mean ± standard deviation.

Abbreviations: CCC, concordance correlation coefficient; FL, false lumen; TL, true lumen.

Of all six aortic locations, the measurements of aortic arch total diameter showed the largest mean difference (1.09 mm) and largest SD of the mean difference (2.58 mm; Figure 3B). Aortic arch agreement scores for diameter and area measurements were overall low, with the poorest agreement in the measurements of the true and FL maximal diameter (CCC = 0.886 and 0.820) (Figure 3C and Table 3). The total aortic area agreement score at the level of the aortic arch was lowest compared with all other locations, with a moderate CCC of 0.907 (Table 3).

**Table 3:** Interobserver agreement for preoperative and postoperative aortic arch up to infrarenal aortic measurements

	Mean ± SD	95% limits of agreement	CCC	CCC agreement
Mid aortic arch				
Total diameter, mm	1.09 ± 2.58	−3.97, 6.16	0.786 (0.563, 0.903)	Poor
TL max diameter, mm	−0.49 ± 2.98	−6.32, 5.35	0.714 (0.433, 0.870)	Poor
FL max diameter, mm	−0.69 ± 4.10	−8.63, 7.25	0.626 (0.207, 0.865)	Poor
Total area, cm^2^	0.45 ± 1.29	−2.08, 2.98	0.775 (0.543, 0.899)	Poor
TL area, cm^2^	0.02 ± 0.80	−1. 55, 1.58	0.864 (0.701, 0.942)	Fair
FL area, cm^2^	0.43 ± 1.38	−2.28, 3.14	0.742 (0.684, 0.877)	Poor
Proximal descending aorta				
Total diameter, mm	0.99 ± 2.35	−3.62, 5.60	0.918 (0.812, 0.966)	Moderate
TL max diameter, mm	−0.05 ± 1.76	−3.51, 3.40	0.911 (0.791, 0.964)	Moderate
FL max diameter, mm	0.17 ± 2.45	−4.64, 4.97	0.904 (0.776, 0.961)	Moderate
Total area, cm^2^	0.59 ± 1.02	−1.42, 2.59	0.937 (0.855, 0.974)	Moderate
TL area, cm^2^	0.03 ± 0.49	−0.93, 0.99	0.956 (0.896, 0.981)	Substantial
FL area, cm^2^	0.56 ± 1.06	−1.52, 2.63	0.924 (0.825, 0.986)	Moderate
Mid descending aorta				
Total diameter, mm	0.97 ± 1.77	−2.51, 4.46	0.882 (0.737, 0949)	Fair
TL max diameter, mm	−0.05 ± 1.22	−2.44, 2.34	0.889 (0.744, 0.954)	Fair
FL max diameter, mm	1.02 ± 2.32	−3.52, 5.56	0.840 (0.660, 0.928)	Poor
Total area, cm^2^	0.45 ± 0.57	−0.67, 1.57	0.928 (0.842, 0.967)	Moderate
TL area, cm^2^	−0.17 ± 0.26	−0.53, 0.49	0.984 (0.961, 0.994)	Substantial
FL area, cm^2^	0.47 ± 0.61	−0.74, 1.67	0.940 (0.867, 0.974)	Moderate
Distal descending aorta				
Total diameter, mm	0.66 ± 0.88	−1.07, 2.39	0.923 (0.829, 0.966)	Moderate
TL max diameter, mm	−0.10 ± 1.16	−2.37, 2.17	0.892 (0.769, 0.951)	Fair
FL max diameter, mm	0.66 ± 1.18	−1.65, 2.97	0.874 (0.724, 0.944)	Fair
Total area, cm^2^	0.34 ± 0.36	−0.36, 1.05	0.909 (0.808, 0.958)	Moderate
TL area, cm^2^	−0.05 ± 0.35	−0.73, 0.63	0.923 (0.823, 0.968)	Moderate
FL area, cm^2^	0.4 ± 0.54	−0.66, 1.45	0.921 (0.823, 0.966)	Moderate
Infrarenal aorta^a^				
Total diameter, mm	0.26 ± 1.09	−1.87, 2.39	0.925 (0.806, 0.972)	Moderate
TL max diameter, mm	−0.49 ± 1.88	−4.17, 3.18	0.886 (0.703, 0.958)	Fair
FL max diameter, mm	0.58 ± 1.09	−1.56, 2.71	0.885 (0.679, 0.962)	Fair
Total area, cm^2^	0.21 ± 0.30	−0.38, 0.79	0.936 (0.817, 0.978)	Moderate
TL area, cm^2^	−0.19 ± 0.59	−1.34, 0.97	0.973 (0.925, 0.990)	Substantial
FL area, cm^2^	0.25 ± 0.33	−0.40, 0.90	0.936 (0.826, 0.977)	Moderate

*Note*: Continuous data are presented as the mean ± standard deviation.

Abbrevaitions: CCC, concordance correlation coefficient; FL, false lumen; TL, true lumen.

^a^ Eight missing patients because of missing subdiaphragmatic data.

For total diameter measurements in the proximal descending aorta, the SD of the mean difference was 2.35 mm and limits of agreement were exceeding the a priori acceptable differences (Figure 3D). In all locations in the descending aorta, diameter measurements revealed a lower agreement score compared with area measurements (Table 3). The distal descending thoracic aorta showed the lowest mean difference and lowest variability for diameter and area assessment (Table 3).

### 3.3 Ellipticity

The average ellipticity index at the six locations for TL and FL were defined (Table 4). The index values were higher than two in almost all regions of the aorta, suggesting no circularity for both the TL and the FL. The highest ellipticity index with the largest SD was present in the aortic arch FL; 4.76 ± 4.97. No correlation was found between the variability of the measurements and ellipticity (*P* = .225). However, a significant negative correlation was found between ellipticity and interobserver agreement (*R* = −0.693, *P* = .026), suggesting that it is more difficult to measure a noncircular aortic geometry.

**Table 4:** Preoperative and postoperative combined ellipticity for unrepaired measured aortic regions along with agreement scores and variability

		Mean ellipticity ± SD	CCC	Variability (average SD from diameter measurements)
Aortic arch	TL	1.68 ± 0.46	0.761	3.81
	FL	4.76 ± 4.97	0.594	4.92
Proximal descending	TL	2.18 ± 1.50	0.942	1.41
	FL	2.03 ± 0.46	0.880	4.08
Mid descending	TL	2.69 ± 1.62	0.921	2.26
	FL	2.05 ± 1.44	0.853	3.35
Distal descending	TL	2.65 ± 1.54	0.910	1.74
	FL	1.92 ± 0.64	0.879	1.62
Infrarenal	TL	2.24 ± 1.20	0.931	0.66
	FL	2.24 ± 1.23	0.909	0.82

*Note*: Continuous data are presented as the mean ± standard deviation.

Abbreviations: CCC, concordance correlation coefficient; FL, false lumen; TL, true lumen.

### 3.4 Volumetric agreement

Volumetric assessment yielded a moderate agreement score for total, TL and FL volume of the preoperative and postoperative CT measurements, with a CCC ranging from 0.908 to 0.941. The mean difference of the total volumetric measurement was 19.20 ± 14.52 mL with 95% limits of agreement of −9.26 to 47.66 mL. The FL volume showed similar variability and 95% limits of agreement of −17.77 to 38.43 mL. The variability was lower in the TL volume measurements with a mean difference of 9.07 ± 4.79 mL.

## 4 DISCUSSION

Morphologic CTA measurements are the main source of information for the assessment of aortic remodeling following acute TAAD. Measurement errors may have an important impact on patient‐specific decision making. However, in the previously reported studies providing predicting factors for adverse aortic remodeling and aneurysmal formation, the reproducibility of various morphologic assessments was not assessed.^4^, ^5^, ^18^, ^19^, ^20^, ^21^, ^22^, ^23^, ^24^, ^25^, ^26^, ^27^, ^28^, ^29^ In the present study, we aimed to provide a comparison of the reproducibility of two‐ and three‐dimensional morphologic measurement techniques.

We summarize our results as follows:

(1) In postoperative TAAD patients, all luminal area measurements are more reproducible than luminal diameter measurements.
(2) In these patients, measurement of the aortic arch shows lowest agreement in comparison to other aortic sites.
(3) And volumetric measurements are not more reproducible than either regional diameter or area measurements.

To the best of our knowledge, this paper describes the first proposed systematic and validated aortic measurement workflow in early DeBakey type I aortic dissections. Our results show that, if a standardized workflow is used, total diameter, area, and volumetric measurements of the thoracic aorta from CTA are reproducible. However, the separate luminal diameter measurements were less reproducible than luminal area measurements. This finding is of important clinical relevance for the assessment of growth in aortic dissections and therefore the timing of early intervention. Moreover, the sizing of stent‐grafts in the case of early endovascular additional repair can be heavily misguided by simple diameter measurements, whereas area measurements may provide a more holistic approach, enabling adequate endovascular intervention. Given the variable morphology of the luminal dimensions in aortic dissection, Sailer et al^30^ proposed to use the circumferential extent of the FL, reflecting the proportion of aortic wall circumference that is characterized by reduced thickness and strength. The study of Sailer et al was performed in TBAD patients and has not yet been validated in other populations of aortic dissection, including postrepair TAAD patients. We did not include this method in this study, as we were interested in the most commonly performed clinical measurements described in predictor studies. The radial displacement of the aorta is known to be nonisometric throughout the cardiac cycle, however, routine clinical CTA data only contains static image data.^31^ Area measurements may account for these variations in deformations better than a single maximal or minimal diameter. We, therefore, elected to include area measurements. To assess the luminal differences in TAAD morphology, an ellipticity index was acquired. Rylski et al^32^ defined circularity as an ellipticity index of less than 1.1. However, our results show that the TL and FL cannot be assumed to be circular in the early phase, since our lowest mean ellipticity index per region was 1.68 with a total range of 1.02 to 21.83. We showed that area measurements have a higher agreement. We thus hypothesize that higher ellipticity index may correspond to less reproducible diameter measurements. Furthermore, we expect the area measurements to be less affected by this loss in reproducibility. If area measurements are not feasible, then circumferential measurements should ideally be used.

**Figure 4:**
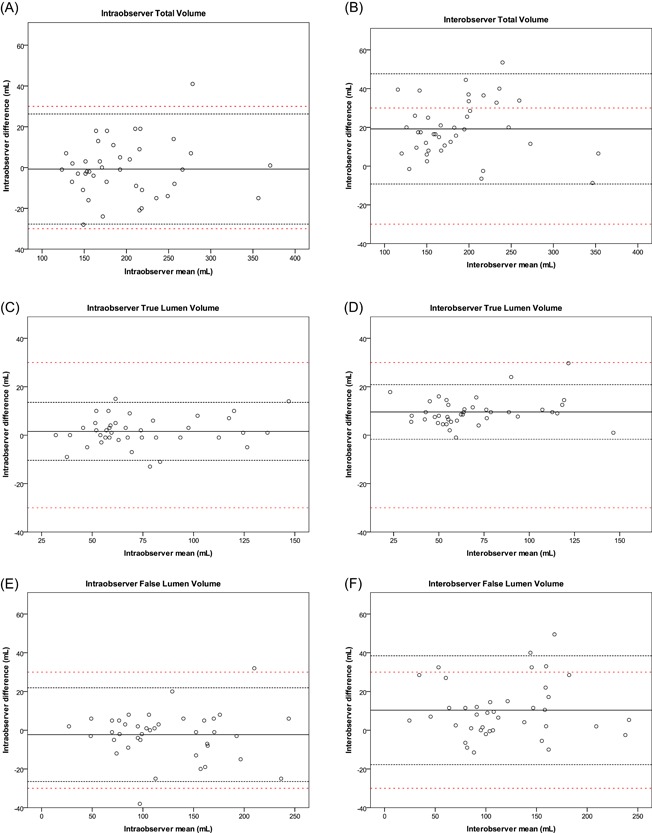
Intraobserver and interobserver Bland‐Altman plots for preoperative and postoperative (A, B) total volumes, (C, D) true lumen volumes, and (E, F) false lumen volumes. The solid black line represents the mean of all measurements paired. The black close dotted lines represent the limits of agreement and the red double spaced dotted line represents the a priori defined acceptable differences. The limits of agreement are defined within the range of the a priori acceptable differences when the black dotted lines both do not exceed the red dotted lines

The CL proved useful for reliable diameter and area measurements. It is argued that a separate FL CL may provide more accurate FL assessment.^15^ The CL through the FL may, however, be harder for automated software to generate and clinical application could lead to comparison of an automated TL CL with a manual FL CL. This should in our opinion be avoided, as this will introduce an additional degree of variation to the analysis. Furthermore, the measurement of distance along the CL will often disagree when using a separate CL for the TL and FL. For volumetric measurements, we did not use a manually generated CL, but a semiautomated vessel filling tool. The borders of the volumes were set by anatomical landmarks (origin of the LSA and celiac trunk). This may account for the lower variability of the volume assessments as compared with the diameter and area assessments (Figures 2, 3, 4). Essentially, volumetric measurements provide more data points compared with two‐dimensional diameter or area measurements. This may be particularly useful for assessment of eccentric aortic dilation during follow‐up, although unlike luminal area or diameter measurements performed at specific levels, changes in volume do not reveal the location of growth. Although volumetric growth measurements demonstrated acceptable reproducibility, the agreement over time was no better than diameter or area measurements.

Previous literature has reported lower variability of total volumetric and FL volumetric assessment in aneurysm and TBAD.^15^, ^33^ In these reports, the volumetric assessment was performed by manual or semiautomated delineation of the outer contour of the volumetric segment. Additionally, the volumetric assessment in aortic aneurysms shows lower variability, underlining the challenge of volumetric assessment in aortic dissection.^33^ In our study, we used a vessel growth tool that semiautomatically filled the vessel from the center of the lumen, which given that FL enhancement is often low‐level and heterogeneous. This explains the high FL volumetric measurement variability compared with the previous reports, as well as the acceptable measurement variability in the TL.

Early TEVAR is an evolving treatment strategy for type I dissections. The timeframe within which an assessment of early aortic growth would be desirable to allow for maximal aortic remodeling is well aligned with the timing of our measurements (ie, 3 months postoperative). In our opinion, it is imperative to assess the reproducibility of a measurement paradigm that uses serial measurements.

### 4.1 Limitations

We have a relatively small sample size, however, there was appropriate statistical power based on our a priori calculations. We compared the preoperative and postoperative CT scans and were not able to completely account for the effects of preoperative and postoperative differences in FL contrast enhancement that could affect the assessment of measurement variability. However, this scenario reflects the actual clinical challenge of determining FL growth at the earliest possible time point to allow for early intervention. Furthermore, it could be argued that we did not assess the association of FL growth with prior or subsequent events, although as we stated, performing a formal assessment of measurement technique was the focus of the paper and determining growth and outcomes was beyond the scope of this paper. A final limitation is arguably the human error introduced by manually segmenting the CL, diameters, areas, and volumetric measurements. In the near future, machine‐learning will have the potential to improve software intelligence, in order to distinguish thrombus, low‐flow lumen area, calcified plaque, aortic wall, and surrounding tissue from one another.

We realize that there are more morphologic characteristics known in the literature to evaluate the aorta. In this study, we studied the most common clinically and scientifically used types and sites of aortic measurement. Comparing our outcomes to other described measurement protocols is beyond the scope of this study.

Entering the deep‐learning age, we wish to stress that two‐dimensional analysis limits accurate assessment. It seems suboptimal not to use all available imaging data, as most image processing software packages have three‐dimensional tools which can provide important information if appropriately processed. The current study demonstrates that volumetric measurements yield acceptable intraobserver and interobserver variability, but perform worse than diameter and area measurements and need to be automated as much as possible to avoid human error and reduced reproducibility. Novel volumetric measurement techniques, such as vascular deformation mapping^34^ may reduce the observer variability of aortic morphology over time, aiding identification of early adverse remodeling, and selection of patients who would benefit from early TEVAR. Up till now this technique is not commonly used and should also be validated in a dissection population.

## 5 CONCLUSION

Commonly used methods of measuring aortic morphology were evaluated for intraobserver and interobserver reproducibility, before and after open surgical repair of acute TAAD. Overall observer agreement is acceptable in total diameter, total area, and volumetric measurements in early TAAD patients. TL and FL diameter measurements have a lower observer agreement, particularly in the aortic arch and the proximal descending aorta. In these locations, area measurements were more reproducible. While diameter measurements are most commonly used to assess aortic enlargement over time, the present study demonstrated that area measurements provide a more reproducible assessment of luminal morphology in patients with a TAAD. The addition of luminal area and possibly volumetric measurements to the standard diameter‐based assessment of aortic dimensions in patients with TAAD may significantly improve the reproducibility of aortic growth measurements, and therefore alter clinical decision making in specific cases.

## CONFLICT OF INTERESTS

The authors declare that there are no conflict of interests.

## AUTHOR CONTRIBUTIONS

IBH: concept/design, data collection, data measurements, data analysis/interpretation, drafting article, and statistics. TMJvB: concept/design, data measurements, data interpretation, drafting article, and critical revision of article. NSB: concept/design, control of data measurements, data interpretation, drafting article, and critical revision of article. FLM: drafting article and critical revision of article. JAvH: critical revision of article. HJP: critical revision of article.
